# Supervised deep learning-based paradigm to screen the enhanced oil recovery scenarios

**DOI:** 10.1038/s41598-023-32187-2

**Published:** 2023-03-25

**Authors:** Rakesh Kumar Pandey, Asghar Gandomkar, Behzad Vaferi, Anil Kumar, Farshid Torabi

**Affiliations:** 1 Department of Petroleum and Energy Studies, School of Engineering and Technology, DIT University, Dehradun, India; 2 Department of Chemical Engineering, Shiraz Branch, Islamic Azad University, Shiraz, Iran; 3 Department of Advanced Calculations, Chemical, Petroleum, and Polymer Engineering Research Center, Shiraz Branch, Islamic Azad University, Shiraz, Iran; 4 Director, Tula’s Institute, Dehradun, 248001 India; 5 Faculty of Engineering and Applied Science, University of Regina, Regina, SK S4S 0A2 Canada

**Keywords:** Energy science and technology, Engineering

## Abstract

High oil prices and concern about limited oil reserves lead to increase interest in enhanced oil recovery (EOR). Selecting the most efficient development plan is of high interest to optimize economic cost. Hence, the main objective of this study is to construct a novel deep-learning classifier to select the best EOR method based on the reservoir’s rock and fluid properties (depth, porosity, permeability, gravity, viscosity), and temperature. Our deep learning-based classifier consists of a one-dimensional (1D) convolutional neural network, long short-term memory (LSTM), and densely connected neural network layers. The genetic algorithm has been applied to tune the hyperparameters of this hybrid classifier. The proposed classifier is developed and tested using 735 EOR projects on sandstone, unconsolidated sandstone, carbonate, and conglomerate reservoirs in more than 17 countries. Both the numerical and graphical investigations approve that the structure-tuned deep learning classifier is a reliable tool to screen the EOR scenarios and select the best one. The designed model correctly classifies training, validation, and testing examples with an accuracy of 96.82%, 84.31%, and 82.61%, respectively. It means that only 30 out of 735 available EOR projects are incorrectly identified by the proposed deep learning classifier. The model also demonstrates a small categorical cross-entropy of 0.1548 for the classification of the involved enhanced oil recovery techniques. Such a powerful classifier is required to select the most suitable EOR candidate for a given oil reservoir with limited field information.

## Introduction

Enhanced oil recovery (EOR) helps optimize the recovery factor to increase the returns from oil and gas projects^1–3^. Increasing oil prices create concern about future energy resources and increase interest in enhanced oil recovery in the world^4,5^. EOR projects are often expensive and have high initial costs than traditional secondary projects^6^. An inappropriate recovery project may lead to permanent damage in the reservoirs and increases financial losses. These analyses comprise laboratory tests and progress through reservoir characterization and simulation, design, and implementation of pilot tests to the final design and implementation of the full field project. Moreover, all the above-mentioned phases involve investments that can be risky if not properly supported by a preliminary cost-efficient screening phase. Hence, a key element of the decision-making approach is, first and foremost, the assessment of the EOR potential for a target reservoir. This is the critical goal accomplished by the practice of EOR screening, which is meant to provide the first metric to be employed for risk reduction with modest capital investment.

Therefore, a reliable and precise enhanced oil recovery screening method is desirable to develop depleting reservoirs. A literature review indicates that there are generally two techniques for EOR screening: (1) conventional EOR screening (CEORS) and (2) advanced EOR screening (AEORS)^7–9^. The CEORS technique considers several predefined screening parameters to indicate the likelihood of successful implementation of each EOR technique. These parameters usually cover the reservoir fluid and rock properties (such as oil saturation, API gravity, layer thickness, formation type, permeability, viscosity, salinity, temperature, and depth) for successful EOR methods^10,11^. These proposed standards were achieved by analyzing the successful EOR projects performed before 1997^10^. Additionally, other factors such as available reserve and implementation costs have a tangible impact on the proposed criteria. These parameters have been extensively used in EOR screening for many years and researchers hardly tried to improve/ update them. Al-Adasani and Bai^12^ reviewed EOR projects conducted since 1998 and improved Taber et al.^10^ proposed principle. Mashayekhizadeh et al. integrated several major screening criteria and produced a set of realism criteria for each EOR technique^13^. Zhang et al. proposed a graphical screening index by analysis of the many enhanced oil recovery projects based on the statistical parameters^14^. Jensen et al. considered CEORS in the Ekofisk field and the results indicated that the water alternating gas injection (WAG) and air injection scenarios are the most suitable EOR methods^15^. Alvarado and Manrique highlighted that the notable limitation of conventional methods is that they only provide a “go/no go” response, without additional details on EOR strategies performed in similar fields^16^. On the other hand, advances in computer science have created a good chance for an alternative approach. In the last decade, computer-aided technology has upgraded EOR screening approaches. The reservoir rock and fluid properties and also the successful implementation of EOR methods play an important role in this approach. This approach was gently extended as AEORS. Similar with other research fields^17^, machine learning methods are also applied to handle the EOR screening^18,19^. Artificial intelligence strategies, including artificial neural networks (ANN)^18,19^, expert systems^20,21^, fuzzy inference^22^, and Bayesian Networks^23,24^ have already been engaged in the EOR classification task. The earliest studies of AEORS were performed by Alvarado et al.^25^. They considered 290 EOR projects around the world and applied dimensionality reduction and clustering methods to create an expert map for choosing a suitable EOR method^25^. Research conducted by Lee et al. includes training an ANN model using 230 successful enhanced oil recovery scenarios to identify the most suitable EOR scenario for candidate reservoirs^26^. In addition, Zerafat et al. integrated the criteria proposed by Taber et al. using 1098 EOR scenarios and developed a Bayesian Belief network to predict the appropriate EOR methods^24^. Parada and Ertekin used a commercial reservoir simulator to collect the data needed to accomplish the ANN train^27^. They proposed a new approach for EOR screening and predicting the performance of enhanced oil recovery scenarios^27^. Several similar studies were also carried out in this field and many machine learning techniques were checked to find an intelligent tool for the EOR screening. Khazali et al. recently trained a fuzzy decision tree-algorithm using 548 successful EOR projects around the world to indicate the screening rules^28^. Babushkina et al. define and investigate analogy by applying a k-Means clustering method on the 6-dimensional space of reservoir rock and fluid properties^29^. The EOR potential of a target field is estimated by interpolation of the recovery factors associated with the (eventually different) EOR techniques of projects belonging to the same cluster^29^. Also, Trujillo et al.^30^ combined conventional and advanced approaches to rank the available database according to a similarity score^10,11^. This approach has made it possible to identify EOR techniques with high potential for application in Colombia’s oilfields.

Consequently, careful and detailed preliminary studies must be performed to reduce uncertainty and minimize the risk of failure of the EOR screening process.

New classes of intelligent techniques, namely deep-learning framework^31^, deep reinforcement learning^32,33^, deep belief network^34^, dual-graph attention convolution network^35^ are recently suggested to monitor (modeling, control, as well as classification) the behavior of even complicated problems. Therefore, the main problem addressed in this work involves selecting the most suitable EOR technique for the target reservoir using a novel deep learning-based classifier. This novel classifier consists of LSTM (long short-term memory), 1D CNN (one-dimensional convolutional neural network), and densely connected neural network (DNN) layers. In addition, the genetic algorithm (GA)^36^ has been used to systematically adjust the hyperparameters of the classifier. The proposed classifier requires a minimum amount of information (i.e. depth, porosity, permeability, oil gravity, viscosity, and temperature) to rank the potential EOR scenarios and suggest the best one. Such a powerful tool can reduce the cost associated with field trials and assist in EOR method selection with greater confidence.

## Data description

The data from 735 real-field EOR projects applied on carbonate, sandstone, unconsolidated sandstone, and conglomerate reservoirs in more than 17 countries have been collected from the literature and used for developing the deep learning-based classifier. This information includes porosity (%), depth (ft), oil gravity (API), permeability (md), viscosity (cP), and temperature (°F) as the independent variables. In addition, the applied EOR scenarios including water flooding (Class 0), CO_2_ flooding (Class 1), hydrocarbon flooding (Class 2), water alternating gas (Class 3), polymer flooding (Class 4), surfactant flooding (Class 5), thermal recoveries such as steam flooding and in-situ combustion (Class 6) are the targets that must be identified.

**Figure 1:**
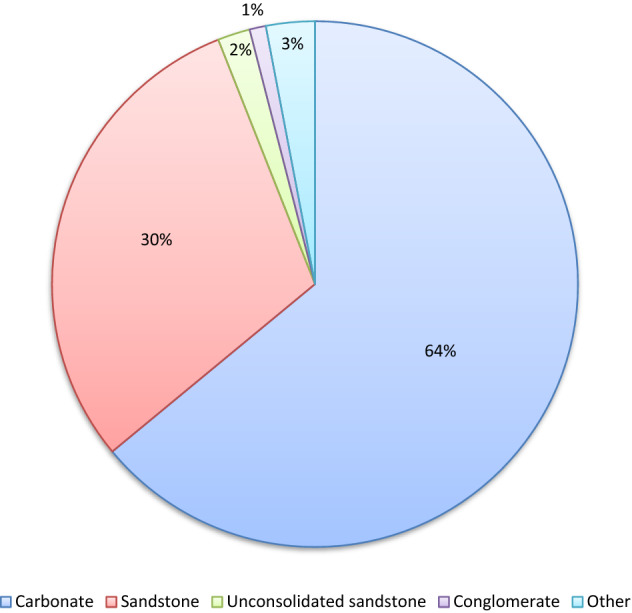
The distribution of EOR methods based on reservoir lithology.

**Figure 2:**
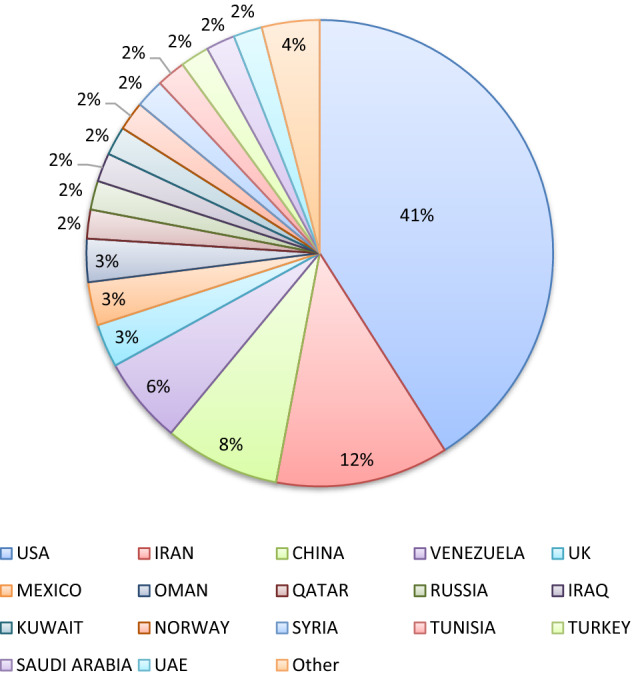
Total available data distribution by country.

Figure 1 presents the distribution of the reservoir lithology available in the collected real-field EOR projects. This figure shows the carbonate and conglomerate reservoirs with the maximum and minimum numbers of the EOR operations in the available database. Furthermore, Fig. 2 differentiates the EOR projects based on the location where they are executed. It can be seen that the collected databank includes the EOR information of more than 17 countries all around the world.

### Data processing

During data pre-processing, the z-score normalization (Eq. (1)) has been applied to scale the input feature^37^.

1 $$NV\, = \,\left( {AV\, - \,\mu } \right)/\sigma ,$$

where AV and NV represent the actual and normalized values of a variable. In addition, μ and σ signify the mean and standard deviation of the variable.

The numerical labels (0 to 6) representing the different EOR methods have been converted to a binary matrix in the current study.

## Deep learning-based paradigm

As explained earlier, this work aims to apply a linearly stacked hybrid three-layered deep-structured network consisting of 1D CNN, LSTM, and DNN to screen EOR methods based on reservoir rock and fluids properties and temperature.

**Figure 3:**
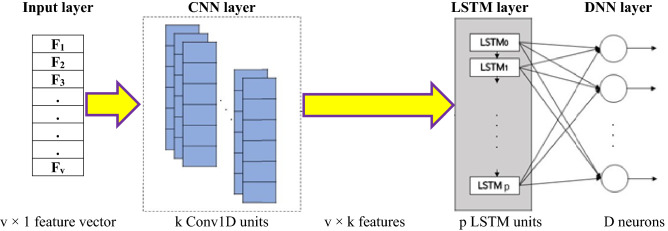
Structure of the hybrid three-layered deep-structured classifier.

Figure 3 presents the general structure of the hybrid classifier used in this study. The numerical value of the normalized independent variables (v × 1 vector) enters into 1D CNN for feature learning^38^. The CNN has k filters of size R1 × 1 that are convolved with the input matrix to produce k feature maps. The rectified linear unit (ReLU) activated CNN gives the output of shape v × k. The LSTM layer with p units and a hyperbolic tangent (Tanh) activation function^39^ provides feedback connection to carry forward the relevant information. Finally, the LSTM output delivers to the DNN layer with D neurons and a softmax activation function to provide the final classification results.

## Results and discussions

### Data distribution

The available dataset (735 samples) has been split into three nonoverlapping groups, i.e. training (90%, 661 samples), validation (7%, 51 samples), and testing (3%, 23 samples). The first group includes samples that help to adjust the tunable parameters of the classifier and accomplish the learning stage. On the other hand, the validation group is used to evaluate the model’s performance during the training step. The last group has been applied to assess the classification performance of the trained model against some unseen samples and monitor its generalization ability.

### Model architecture

Since the number of independent variables and EOR classes dictates by the investigated problem, it is only necessary to regulate the number of units in the CNN and LSTM layers. The GA which provides an optimal or near-optimal solution of a pre-defined objective function from the problem space^40^, has been utilized in this work to tune these two hyperparameters. The population was initialized using random sampling, and the GA evolved over 500 generations using tournament selection, one-point crossover, and mutation operators. The GA minimizes the categorical cross-entropy (CCE) function to obtain optimal hyperparameters’ values, including the number of filters in the CNN layer (Conv1D units) and the number of units in the LSTM layer (LSTM units). Table 1 provides the bounds of the search space which are utilized during GA optimization.

**Table 1:** Search space for the variables during GA optimization.

Hyperparameter	Abbreviation	Range of investigation
Number of filters in the CNN layer	Conv1D units	[1, 500]
Number of units in the LSTM layer	LSTM units	[1, 500]

**Figure 4:**
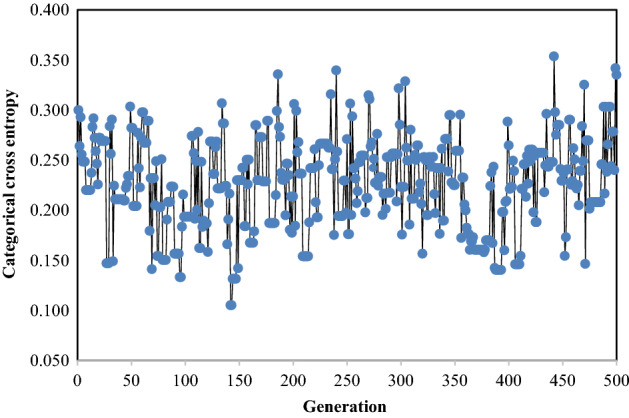
Variation of the CCE by the GA generation.

Figure 4 introduces a variation of the CCE by the GA generation when the number of filters in the CNN layer and units in the LSTM layer is changed in the predefined ranges. This figure shows that the minimum CCE of 0.1050 is achieved in the 143rd generation. This minimum CCE is associated with the Conv1D, and LSTM units as 349 and 60, respectively.

Table 2 summarizes the key characteristics of the structure-tuned deep learning classifier by the GA.

**Table 2:** The key features of the structure-tuned deep learning-based classifier by the GA.

Layer	Output shape	Number of parameters	Activation function
CNN	(None, 6, 349)	698	ReLU
LSTM	(None, 60)	98,400	Tanh
DNN	(None, 7)	427	Softmax

### Structure-tuned classifier

The previous analysis approves that the hybrid sequential model with three layers (i.e. CNN with 349 units and LSTM with 60 units) is the best classifier to select the most efficient EOR scenario for a considered oil reservoir. This classifier only needs to receive the normalized matrix of the six independent features to rank the EOR classes.

### Evaluation model performance

#### Numerical analysis

The CCE and accuracy indices have been applied to evaluate the classifier’s performance. The mathematical expressions of these indices are shown in Eqs. (2) and (3)^41^.

2 $$CCE\, = \,\left( {1/N} \right)\, \times \,\sum\nolimits_{k = 1}^{N} {\left[ {n_{k} \, \times \,\log \,\left( {\overline{n}_{k} } \right)\, + \,\left( {1 - n_{k} } \right)\, \times \,\log \,\left( {1 - \overline{n}_{k} } \right)} \right]}$$

3 $$Accuracy\,\left( \% \right)\, = \,100\, \times \,\frac{Numbers\,of\,the\,true\,detection}{{Numbers\,of\,true\,and\,false\,detection}},$$

where N is the number of the data sample; $n_{k}$ and $\overline{n}_{k}$ present the kth actual and estimated values.

Table 3 summarizes the numerical value of the CCE as well as the accuracy of the designed deep learning-based classifier in the training, validation, and testing stages. It should also be noted that our deep learning classifier identified the correct EOR scenario of 735 field examples with an overall accuracy of 95.92 and CCE = 0.1548.

**Table 3:** The numerical values of the achieved CCE and accuracy by the developed classifier.

Classification stage	CCE	Accuracy (%)
Training	0.1050	96.82
Validation	0.6082	84.31
Testing	0.5914	82.61

#### Performance analysis by the confusion matrix

The confusion matrix^42^ is a well-established graphical technique to easily assess the reliability of a classifier. This technique reveals the number of correct as well as incorrect identifications of each involved class. Indeed, the records located in the diagonal cells indicate the number of correct identifications for the involved classes. Furthermore, other records in the confusion matrix are incorrect identifications.

**Figure 5:**
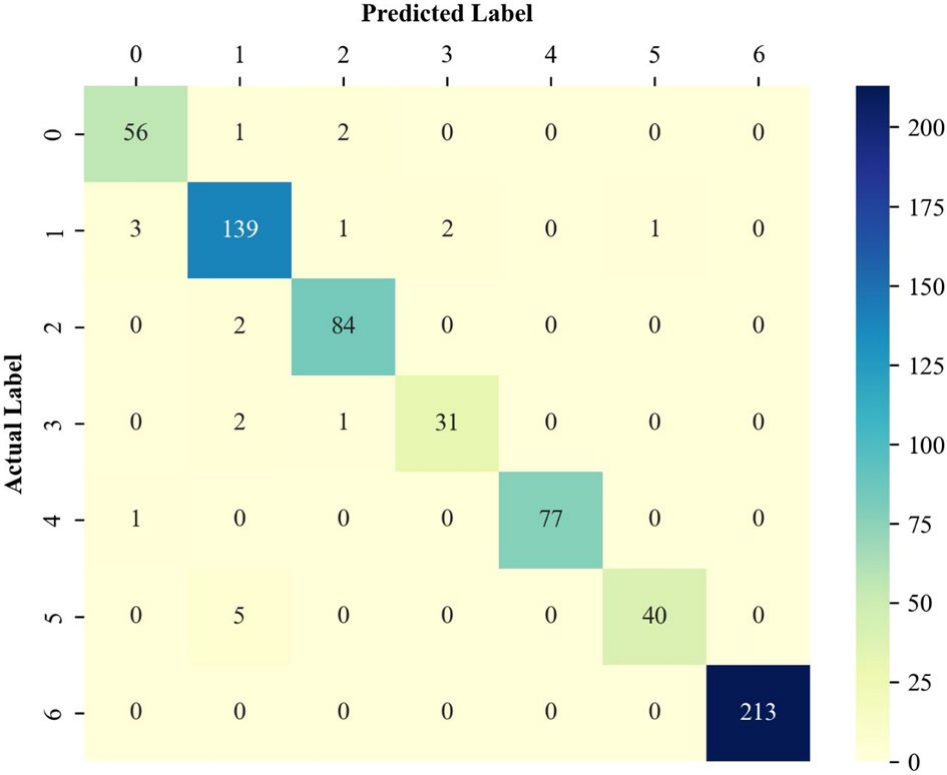
The confusion matrix of the designed model related to the training data classification.

**Figure 6:**
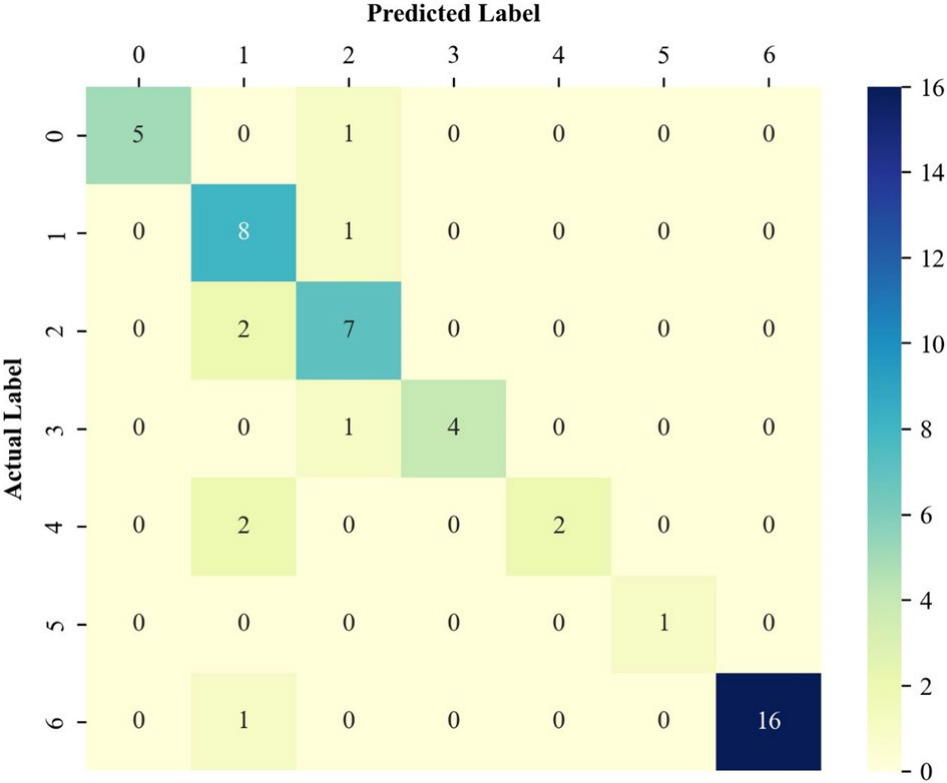
The confusion matrix of the designed model related to the validation data classification.

**Figure 7:**
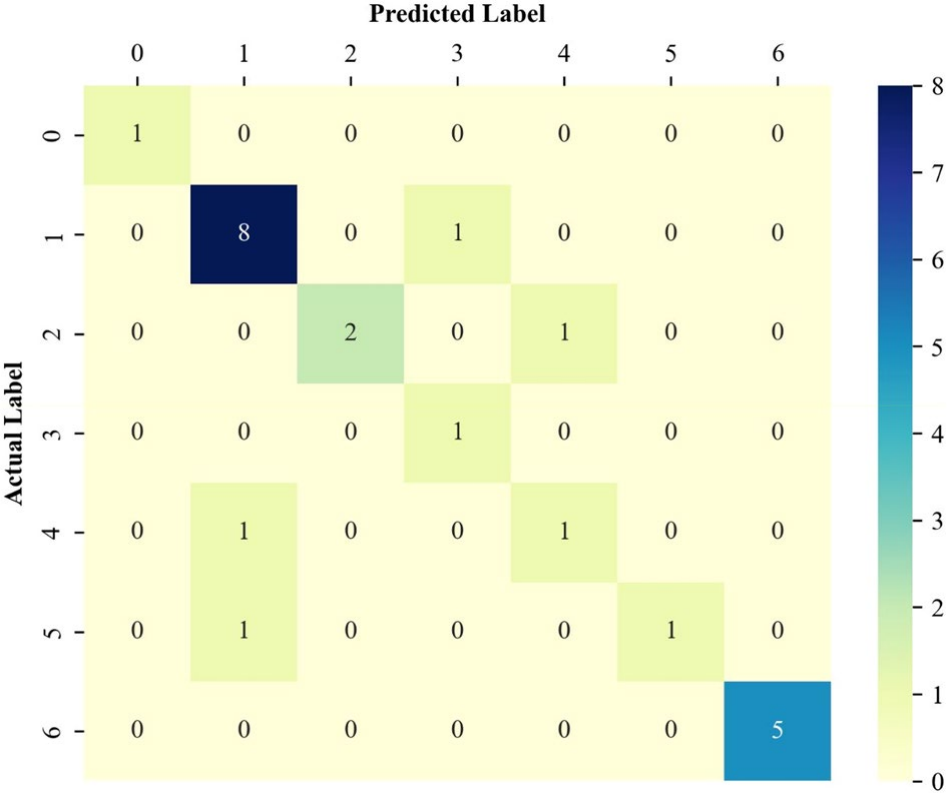
The confusion matrix of the designed model related to the testing data classification.

The confusion matrices associated with the training, validation, and testing stages are presented in Figs. 5, 6, and 7, respectively. Figure 5 clarifies that the proposed deep learning model correctly identifies 640 out of 661 EOR scenarios in the training stage. Furthermore, the designed classifier shows outstanding performance in the correct identification of both the validation and testing groups. Indeed, it correctly distinguishes 43 out of 51 validation samples and 19 out of 23 unseen testing examples.

## Conclusions

This research aims to employ the deep learning-based structure for selecting the most suitable EOR scenario based on the oil reservoir characteristics including depth, porosity, permeability, gravity, viscosity, and temperature. The information on 735 real-field EOR projects collected from the literature has been used to design the considered classifier and monitor its accuracy. The utilized databank includes the EOR scenarios applied on the carbonate, sandstone, unconsolidated sandstone, and conglomerate reservoirs in more than 17 countries. The hyperparameters of the deep learning-based classifier have been tuned by the GA. It was found that the 1D CNN and LSTM layers of the classifier must have 349 and 60 units, respectively. The structure-tuned deep learning classifier identified the correct EOR scenario of 735 field examples with excellent accuracy of 95.92 and a small CCE of 0.1548. Such a reliable tool can easily reduce the cost associated with checking several EOR projects based on the try-and-error procedure.

## Data Availability

All analyzed data in this study collected from the literature are available on reasonable request from the corresponding author (Dr. B. Vaferi).
